# Adverse Childhood Experiences and Hospital-Treated Self-Harm

**DOI:** 10.3390/ijerph15061235

**Published:** 2018-06-11

**Authors:** Seonaid Cleare, Karen Wetherall, Andrea Clark, Caoimhe Ryan, Olivia J. Kirtley, Michael Smith, Rory C. O’Connor

**Affiliations:** 1 Suicidal Behaviour Research Laboratory, Institute of Health and Wellbeing, College of Medical, Veterinary and Life Sciences, University of Glasgow, Gartnavel Royal Hospital, 1055 Great Western Road, Glasgow G12 0XH, UK; Karen.wetherall@glasgow.ac.uk (K.W.); Andrea.clark@glasgow.ac.uk (A.C.); Rory.oconnor@glasgow.ac.uk (R.C.O.C); 2 School of Psychology and Neuroscience, University of St Andrews, South St, St Andrews KY16 9JP, UK; cr487@st-andrews.ac.uk; 3 Center for Contextual Psychiatry, Department of Neuroscience, KU Leuven, 3000 Leuven, Belgium; olivia.kirtley@kuleuven.be; 4 NHS Greater Glasgow and Clyde, Commonwealth House, 32 Albion Street, Glasgow G1 1LH, UK; Michael.Smith2@ggc.scot.nhs.uk

**Keywords:** suicidal behaviour, self-harm, risk factors, adverse childhood experiences

## Abstract

Adverse childhood experiences (ACEs) have been implicated in a range of negative health outcomes in adulthood, including increased suicide mortality. In this study, we explored the relationship between ACEs and hospital-treated self-harm. Specifically, we investigated whether those who had a history of repeat self-harm reported more ACEs than those who had self-harmed for the first time. Patients (*n* = 189) admitted to two hospitals in Glasgow (UK) following first-time (*n* = 41) or repeated (*n* = 148) self-harm completed psychosocial measures. Univariate analyses revealed that those presenting with repeat self-harm reported higher depressive symptoms, anxiety symptoms, intent to die, and ACEs, and lower dependent attachment style. However, only ACEs, along with female gender and depressive symptoms, significantly differentiated between the repeat self-harm group and the first-time self-harm group in the multivariate model. Controlling for all other psychosocial variables, participants who reported 4+ ACEs were significantly more likely to be in the repeat self-harm group as compared to those who experienced 0–3 ACEs. This finding highlights the pernicious effect of exposure to multiple ACEs. Further research is urgently required to better understand the mechanisms that explain this relationship. Clinicians should be aware of the extent of the association between ACEs and repeat self-harm.

## 1. Introduction

Suicide and self-harm are major public health concerns worldwide [1]. Like many other European countries, suicide is the leading cause of death among people aged 15–34 years in Scotland, and one of the main causes of premature death in men [2]. Although studies have identified a range of factors which increase the risk of suicide (e.g., depression, entrapment, lack of social support, social disadvantage) [3], for the most part, these factors are too generic. Consequently, it remains difficult to identify specific individuals within high risk groups who are more likely to take their own lives than others [4,5,6]. A key challenge for the field, therefore, is to better understand the characteristics of people with suicidal behaviour, so as to be able to respond to their needs and minimise the risk of repetition of self-harm and suicidal behaviour.

To date, the most consistent predictor of a future suicide attempt is history of a previous suicide attempt [7], or having engaged in non-suicidal self-injury (NSSI) [8]. Previous research suggests that around half of those who take their own lives have self-harmed in the past [9]. Repetition of self-harm is relatively common in the months following an index episode; about 2–7% will die by suicide in the next 1–9 years [10,11] and people who attend an emergency department following a suicide attempt have a 16.3% increased risk of making another suicide attempt and a 3.9% risk of dying by suicide within 5 years [12]. Indeed, past hospital treatment for any type of self-harm (defined as intentional self-injury regardless of suicidal intent [13,14]), is a strong predictor of future suicide [3,15]. Specifically, individuals who present to hospital with self-harm are 30 times more likely to die by suicide than those in the general population [16]. As yet, we do not fully understand why some people repeatedly engage in self-harm, whereas others engage in self-harm only once.

### Early Life Adversity and Self-Harm

Exposure to adversity early in life has been associated with a range of negative consequences including poor mental health, substance abuse, relationship problems, suicide and self-harm in adulthood [17,18,19]. Early life adversities are often referred to as adverse childhood experiences (ACEs) and they include exposure to domestic violence, physical or sexual abuse, emotional neglect, parental separation, and exposure to a household member’s substance misuse, mental illness, suicide, or imprisonment. Exposure to one or more ACEs is common, and in a recent household survey of adults in England almost half (46.4%) of respondents reported experiencing at least one ACE and just under one-tenth (8.3%) had experienced four or more ACEs [20]. Some research has suggested that ACEs often co-occur [21], for instance, children who experienced childhood sexual abuse were also more likely to have been exposed to verbal or physical abuse and neglect [22].

Exposure to ACEs can have implications for attachment formation and relationships in adulthood [23,24]. Secure attachment develops in the context of a supportive and nurturing environment where the carer provides a “safe haven” for the child to explore the world and is there to manage their distress. Raised in this environment, the child develops the ability to manage their own distress and self-soothe [25]. Abuse and neglect in childhood are associated with the development of maladaptive attachment styles [26]. In turn, insecure attachment styles are often associated with deficits in regulating emotional responses and use of maladaptive emotional regulation behaviours including self-harm [27,28]. Indeed, studies with adolescents who have experienced ACEs have found that NSSI is often employed as a means of emotion regulation [29]. 

As noted elsewhere, the presence of multiple ACEs has been associated with negative health outcomes including physical health problems and higher mortality, as well as increasing the risk of suicide ideation and suicide [17]. Although exposure to four or more ACEs has been found to increase the risk of adult physical illnesses like cancer and heart disease approximately two-fold, it increases the risk of attempted suicide 12-fold, after adjustment for demographic factors [17]. The literature consistently indicates that the greater the exposure to ACEs, the higher the risk of mental health problems. Although much of this research has been conducted in the United States [30] research conducted in European countries echoes these findings and has highlighted that as exposure to ACEs increases, this negatively impacts upon mental wellbeing [20] and is associated with increased risk of suicidal ideation [31]. Additionally, early life trauma is also associated with a blunted stress response among adults with a suicidal history [32].

Despite the compelling evidence, from the United States in particular, the extent to which ACEs are important factors in the aetiology of self-harm in European countries is relatively unknown. Scotland in particular is a good candidate; the country experiences a pervasive health inequality that is more pronounced than its UK counterparts; although psychosocial issues such as drug and alcohol misuse are high, the increased morbidity and mortality rates are still unaccounted for [33]. Indeed suicide rates are higher than in England and Wales [34], and a recent prevalence study indicated higher rates of self-harm and suicide attempts in young adults compared to those found in similar studies conducted in England [35]. In this study, therefore, we explored the relationship between ACEs and episodes of hospital-treated self-harm in a Scottish sample of inpatients. Specifically, we predicted that those who had a history of repeated self-harm (repeat self-harm episode group) would report more ACEs than those who had self-harmed for a first time (first self-harm episode group), and that this would be associated with self-harm history when other established risk factors for self-harm (symptoms of anxiety, depression and attachment style) were controlled for. As multiple reasons often underlie self-harm [36] and a person’s ”desire to die”[37] can vary from moment to moment, we included patients presenting to the hospitals with any form of self-harm. This is consistent with the UK national clinical guidance around the management of self-harm, which defines self-harm as “self-injury or self-poisoning irrespective of the apparent purpose of the act” [38].

## 2. Materials and Methods

### 2.1. Participants

All patients who were admitted to either an acute receiving unit or a general medical ward in two general hospitals in Glasgow via the Emergency Department (Glasgow Royal Infirmary (GRI) and Queen Elizabeth University Hospital (QEUH), Glasgow, Scotland) following an episode of self-harm (International Classification of Diseases (ICD) codes X60-X84, intentional self-harm) between 20 April 2016 and 31 August 2017 were considered eligible for participation in the study. These are the only two general hospitals in Glasgow with emergency departments, and they serve the whole city. The National Health Service (NHS) Greater Glasgow and Clyde serves one-fifth of Scotland’s population (1,137,930 people); Glasgow city makes up 52.4% of this population. Patients were eligible to take part if they were over 18 years of age, and were assessed by a member of the Liaison Psychiatry team at either site following an episode of self-harm. Psychosocial assessment by the Liaison Psychiatry team is standard usual care for any patient admitted to one of the hospitals following self-harm. The nature of this assessment varies as function of the severity and nature of the self-harm presentation. Exclusions included patients being unable to provide written informed consent (e.g., being medically unfit (e.g., currently intoxicated, receiving treatment for injuries) or not competent in English) or if they were actively psychotic, aggressive, or were prisoners.

### 2.2. Measures and Procedure

The majority of participants (80.4%, *n* = 152) were interviewed within two days of their index episode. Potential patients were identified by Liaison Psychiatry staff who established a patient’s medical fitness (i.e., ability to give informed consent) to be approached regarding the study. If patients were medically fit, a member of the Liaison Psychiatry team asked if they were willing to speak to the researcher to find out more about the study. If so, the researcher visited the patient at their bedside to provide further information about the study, answer questions and complete informed consent. Interviews were conducted either at patients’ bedsides or in a private room, depending on patients’ preferences. Participants had the option of completing the questions by themselves, responding via response cards (with researcher reading the questions aloud) or verbal response. Interviews were carried out by members of the research team, who were trained in administering the measures. Almost all of the participants used the response cards to complete the measures. The voluntary nature of the study was emphasised. It was also made clear that non-participation would not interfere with their treatment during or following their stay in hospital. Participants received no incentive to participate in the study.

### 2.3. Demographics

Demographic information including age, gender, marital and employment status and living arrangements was collected directly from the participants and medical records.

### 2.4. Self-Harm History

Self-harm history was established via items adapted from the Adult Psychiatric Morbidity Survey (APMS; [11]) to assess lifetime prevalence of NSSI and suicide attempts. These behaviours were assessed by the following items: “Have you ever harmed yourself without wanting to die, by taking an overdose of tablets or in some other way?” and “Have you made an attempt to take your life, by taking an overdose of tablets or in some other way?”. Self-harm history includes endorsements of either or both of these behavioural items. Participants were also asked to indicate how many times in their life they had engaged in these behaviours.

### 2.5. Suicidal Intent

Participants were asked “Did you intend to kill yourself this time?”. Responses were classified as “yes”, “no” or “don’t know”. This method has been successfully employed in previous hospital studies conducted by our research group [39].

### 2.6. Adverse Childhood Experiences (ACEs)

A 10-item version of the Adverse Childhood Experiences Questionnaire (ACE Questionnaire [17,18]) was used to establish exposure to negative life experiences during the first 18 years of life. The ACEs measure assesses the presence or absence of the following negative experiences; verbal or physical abuse, sexual abuse, and physical and emotional neglect as well as the individual’s exposure to maternal abuse, parental separation, and/or a household member’s substance abuse, mental illness, or incarceration (e.g., “while you were growing up did a parent or adult in the household ever hit you so hard that you had marks or were injured?”). As Felitti and colleagues [17] found that an ACEs score of 4 or more incidents had a marked impact on health outcomes, we dichotomised the ACEs scores into 0 (0–3) versus 1 (4+).

### 2.7. Depressive Symptoms

Recent depressive symptoms were assessed using the 10-item Patient Health Questionnaire (PHQ-9; [40]). The measure comprises 10 items; 9 items assess the presence of the nine Diagnostic and Statistical Manual of Mental Disorders (DSM) criteria for major depressive disorder over the last two weeks (e.g., “Little interest or pleasure in doing things?”) on a 4-point Likert-type scale (0–3) and a standalone item to establish the impact of the symptoms on everyday functioning. The PHQ-9 has been found to be a reliable and valid measure of depression in a variety of populations [40] and is widely used for assessing depressive symptoms in clinical practice [41]. Cronbach’s alpha (α) in our study was good (α = 0.83).

### 2.8. Anxiety Symptoms

Anxiety symptoms were assessed using the 7-item Generalised Anxiety Disorder questionnaire (GAD-7; [42]). This brief measure employs a 4-point Likert-type scale (0 = “not at all” to 3 = “nearly every day”) to assess the extent to which the participant has been feeling nervous, anxious or on edge (e.g., “feeling afraid as if something awful might happen”) in the preceding two weeks. The GAD-7 has been found to be a reliable and valid measure of anxiety [43] and widely used to assess anxiety. The α was acceptable in our sample (α = 0.78).

### 2.9. Attachment Style

The 18-item Revised Adult Attachment Scale - Close Relationships Version (RAA; [44]) was used to assess three dimensions of attachment: (1) ease with closeness and intimacy (closeness: e.g., “I find it relatively easy to get close to people”); (2) worries about abandonment or absence of love (anxiety: e.g., “I often worry that other people don’t really love me”); and (3) feeling others are dependable for support (dependency: e.g., “I know that people will be there when I need them”). It uses a 5-point Likert-type scale to assess how like them each statement is (1 = “not at all characteristic” to 5 = “very characteristic of me”). The RAA has previously demonstrated good psychometric properties [45]. In our sample the overall α was acceptable (α = 0.72), however two of the subscales were relatively low (closeness α = 0.52; anxiety α = 0.46) and one acceptable (dependency α = 0.71).

### 2.10. Ethics

Ethical approval was granted by the West of Scotland Research Ethics Committee (Ref: 16/WS/0014). Participants were provided with written and oral information about the study and all participants provided written informed consent to take part.

### 2.11. Data Analysis

All statistical analyses were conducted using SPSS v.24 (IBM Corp., Armonk, NY). In addition to descriptive statistics, initial univariate binary logistic regressions were used to explore which variables differentiated between those admitted for their first self-harm episode versus a repeat self-harm episode. Those that were significantly different between the groups were then included in a multivariate binary logistic regression to establish which differentiated between the groups when other variables were controlled for. The odds ratios (OR), 95% confidence intervals (CI), and, where applicable, means (Ms) and standard deviations (SDs) are reported for the logistic regression analyses.

#### Missing Data

A missing values analysis was conducted for all variables. Participants were excluded from the main analyses if they had not completed the ACEs measure or the self-harm items (*n* = 9). We investigated whether there were any differences between those who did versus did not complete the ACEs measure/self-harm items. One difference emerged; those who did not complete these measures were significantly older (mean age = 49.4, SD = 12.4) than those who did (mean age = 35.9 years, SD = 13.04; t (196) = 3.05, *p* = 0.003).

Scales were then assessed and if a participant had completed less than 75% of a measure they were classified as incomplete and their data were omitted from the analysis for that scale. Following exclusion of the latter (range *n* = 1 to *n* = 9 across measures), missing data ranged from 0.5% (PHQ-9 and GAD) to 1.6% (RAA) and missing value analyses established that there was no pattern to the items missed on any of the scales. As a result, the missing data were replaced using Expectation-Maximization replacement methods. We did not replace any missing data for the self-harm history and ACE questions.

## 3. Results

### 3.1. Sample and Participant Characteristics

Over the duration of the study 573 potentially eligible individuals were assessed by Liaison Psychiatry. Of these, 220 individuals were discharged before the researcher could approach them; 105 were not well enough to be approached; for 15 it was not appropriate to approach them for the study (i.e., care plans minimizing contact); and 35 declined to take part. A total of 198 individuals consented to take part in the study, however 9 participants were subsequently excluded from analyses as they did not complete the measures.

Of the 189 participants included in the analysis, 128 (68%) identified as female, 60 (32%) identified as male, and one person declined to indicate their gender. The age range of the sample was 18–74 years old (mean age = 35.9, SD = 13.04). The sample was primarily white (*n* = 182, 97.8%); around three quarters (*n* = 149, 78.8%) reported having never been married, and 21.2% were living with a partner, married, or in a civil partnership (see Table 1). With regard to gender differences, women were significantly younger than men (*t* (186) = 2.02, *p* < 0.05), and although there were no gender differences in the total number of ACEs experienced, women were significantly more likely than men to report emotional neglect (X^2^ (1, *n* = 187) = 8.4, *p* = 0.004). Women also scored lower on the dependent subscale of the RAA, indicating they perceive their social support to be less available to them (*t* (186) = 2.2, *p* = 0.03). There were no significant differences in any demographic characteristics of participants recruited between the two hospital sites.

### 3.2. Self-Harm History and ACEs

Over one-fifth of participants (21.7%; 41/189) were recruited following their first episode of self-harm, and 148 (78.3%) participants reported previous engagement in self-harm. The primary presentation for participants was overdose (92.6%, *n* = 175), 2.6% (*n* = 5) presented following self-cutting, and 4.8% (*n* = 9) had engaged in another method of self-harm or mixed methods.

Within the repeat self-harm group, 17% (*n* = 25) reported 1–2 previous episodes of self-harm, 30.6% (*n* = 45) 3–4 previous episodes, and 52.4% (*n* = 77) reported 5 or more episodes. Across our sample 89.4% of participants (*n* = 169) had experienced at least one category of ACE. The variable was dichotomised, with 43.9% reporting 0–3 ACEs and 56.1% reporting four or more ACEs (see Table 2 below).

### 3.3. Univariate Analysis

Univariate binary logistic regressions exploring differences between the first episode group and the repeat self-harm groups are reported in Table 1. The groups did not differ in the majority of demographic characteristics (e.g., education, relationship status and employment) except for gender; with significantly more women comprising the repeat self-harm group than the first self-harm episode group (OR = 2.36, 95% CI = 1.15–4.84, *p* = 0.02).

Differences between the groups were also found on intent to die, with the repeat episode group being 2.5 times more likely to express intent to die associated with their current self-harm episode (OR = 2.50, 95% CI = 1.22–5.03, *p* = 0.01). Those in the repeat self-harm episode group reported higher depressive symptoms (OR = 1.11, 95% CI = 1.05–1.17, *p* = 0.001) and anxiety symptoms (OR = 1.08, 95% CI = 1.01–1.16, *p* = 0.026) than those in the first-time episode group. The repeat and first episode groups differed on the dependent dimension of attachment; those in the repeat self-harm episode group rated feeling that support was less available to them if they needed it compared to the first time group members (OR = 0.95, 95% CI = 0.85–97, *p* = 0.002).

### 3.4. Adverse Childhood Experiences (ACEs)

Of the ten ACEs categories, five differentiated between the groups in univariate analyses (see Table 2 for full details). Significantly more of the participants in the repeat self-harm episode group had experienced verbal/fear of physical abuse (OR = 2.55, 95% CI = 1.22–5.33, *p* = 0.013), physical abuse (OR = 2.95, 95% CI = 1.38–6.34, *p* = 0.005), emotional neglect (OR = 2.88, 95% CI = 1.39–5.94, *p* = 0.004), or had grown up with a family member experiencing mental ill health (OR = 2.30, 95% CI = 1.10–4.79, *p* = 0.03). Additionally, those in the repeat self-harm group were three times more likely to have reported experiencing childhood sexual abuse than those in the first episode group (OR = 3.01, 95% CI = 1.19–7.65, *p* = 0.02).

The groups also differed significantly in the number of ACEs experienced, with the repeat episode group experiencing a higher total number of ACEs than the first episode group (OR = 1.22, 95% CI = 1.06–1.40, *p* = 0.004). As noted, we dichotomised the total number of ACEs (0–3 vs. 4+), and participants in the repeat episode group were over three times more likely to have experienced four or more adverse experiences before age 18 than those in the first-time group (OR = 3.17, 95% CI = 1.53–6.55, *p* = 0.002).

### 3.5. Multivariate Analysis

Next, a multivariate binary logistic regression analysis was conducted to determine the independent effects of each variable in distinguishing between the first time and the repeat self-harm groups. All of the variables that significantly distinguished between the groups in univariate analysis were entered into the model, displayed in Table 3. In the multivariate model, those in the repeat self-harm group experienced significantly higher depressive symptoms (OR = 1.10, 95% CI = 1.00–1.21, *p* = 0.048) than those in the first episode group, as well as being more likely to be female (OR = 2.2, 95% CI = 1.00–4.83, *p* = 0.05). Importantly, those in the repeat self-harm group were 2.4 times more likely to have experienced multiple (4+) ACEs than those in the first episode group (OR = 2.4, 95% CI = 1.05–5.40, *p* = 0.038), when controlling for other demographic and psychosocial variables.

## 4. Discussion

We explored the relationship between ACEs and hospital treated self-harm, and whether the frequency of ACEs was associated with self-harm history. As predicted, we found that those who had a history of repeat self-harm (repeat self-harm episode group) reported significantly more ACEs than those who had self-harmed for the first time (first self-harm episode group). We also found that the association between experiencing multiple ACEs and repeat self-harm remained significant even when other risk factors (e.g., depressive symptoms) were controlled for in multivariate analyses.

Our study suggests that there is an association between the number of ACEs experienced and repeat self-harm. Over 50% of participants in the repeat self-harm group reported exposure to verbal or physical abuse or emotional neglect whilst growing up, compared to around a third of the first self-harm episode group. More participants in the repeat episode group reported experiencing sexual abuse than those in the first self-harm episode group (34.7% vs. 15% respectively). Our findings are similar to those of Bellis and colleagues [46] who found that experiencing childhood physical, sexual, or emotional abuse, witnessing maternal abuse, and living in a household affected by mental illness were all associated with lower levels of mental wellbeing.

Previous research has shown that ACEs often co-occur and that the cumulative effect of ACEs is strongly associated with negative physical and mental health outcomes and increased mortality risk in adulthood [17]. As noted above, individuals who have experienced four or more adversities appear to be at increased risk of repeat self-harm compared to those who have experienced 0–3 ACEs. In our study 62% of the repeat episode group reported experiencing four or more ACEs. In comparison 4 or more ACEs are reported by around 8–10% of respondents in general population studies [17,47].

Adverse experiences during childhood can hinder the development of a secure attachment style which can impact upon subsequent relationships [27,28] and lead to difficulties in regulating emotional responses [29]. Indeed, those in the repeat self-harm group scored lower than those in the first episode group on an established measure of attachment that assessed the extent to which respondents felt secure in their relationships. However, when we evaluated the three subscales of the measure individually, this finding was driven by those in the repeat episode group perceiving lower availability of support. Our findings echo those of previous research, particularly of Bellis and colleagues [46] who found a relationship between ACEs and not feeling close to others operationalised as “always available adult” in childhood [46]. From the univariate analyses, it was evident that those in the repeat self-harm group also reported more symptoms of depression and anxiety and 70% of this group also expressed an intent to end their lives.

### Clinical Implications

Scotland has higher morbidity and mortality rates than other regions of the UK which have not been adequately explained by psychosocial issues such as drug and alcohol misuse. In particular, rates of self-harm and suicide attempts in young adults in Scotland are higher than those reported in other areas of the UK [34,35]. Understanding antecedents of self-harm is paramount to reducing the occurrence of such potentially devastating behaviour. Our finding that those in the repeat episode group were three times more likely to have experienced four or more ACEs highlights the importance of clinicians assessing exposure to ACEs in vulnerable individuals. In short, such individuals are likely to require more targeted clinical intervention.

Although the findings from our study suggest a link between multiple ACEs and repeat self-harm, future research is required to understand this relationship more fully, including determining mechanisms that may explain the association. Extrapolating from the multivariate findings, it appears that symptoms of depression, anxiety and attachment style do not account for the association.

## 5. Limitations and Future Directions

The data reported herein were cross-sectional, which limits the conclusions we can draw in terms of causality or direction of effect. Employing the present study design, it is not possible to determine whether some of those in the “first-episode group” will engage in further self-harm in the future. Prospective studies are needed to understand the utility of ACEs to predict repeat self-harm over time. Another potential limitation is our sample size. The first episode of self-harm group was comprised of 41 participants, and arguably given the likely heterogeneity of this group, there may be considerable statistical noise in the dataset. Our sample size limited the subgroup analysis we were able to carry out. For instance, there could be differences in the clinical profiles of individuals who expressed intent to die, reported no intent or were unsure within the first episode and repeat episode groups. Future studies may investigate differences in these subgroups.

There may be some issues with generalisability of these findings; firstly the sample was overwhelmingly white, which does accurately not reflect the Scottish population which is more mixed [48]. Additionally, it must be noted that our sample of patients requiring inpatient care for physical health problems after self-harm may not be representative of the wider population of people who present at emergency departments with self-harm, namely as those reporting five or more past episodes may have been overrepresented [49].

This may have been in part due to our recording of self-harm history; we recorded any incidents of self-harm reported by participants rather than just hospital-treated self-harm. This means we are unable to report on the medical severity of previous episodes and reporting may vary between individuals. Similarly, this sample includes participants who reported a wide range of previous episodes, with some participants reporting previous episodes that were too numerous to count (in the thousands). Another issue to consider in future research is the issue of recency, as some of our participants’ previous episode(s) may have been many years ago, as compared to the last few weeks or months for others.

Our sample may also be skewed by our recruitment method which required individuals to be referred following assessment by Liaison Psychiatry staff. This procedure was in place as patient welfare is paramount, however, patients were often discharged before the researcher was able to meet them.

Although we controlled for suicidal intent in our analyses, larger studies are required to explore differences between subgroups of people with different histories of suicide attempts and non-suicidal self-harm. Finally, the measure of ACEs, although well validated, only assesses the presence or absence of a limited range of experiences; it is not exhaustive and we did not record further information about the impact of participants’ experiences. Factors identified in more recent work on “extended ACEs” (including community violence, bullying, poverty and discrimination) [50,51,52] are likely to be relevant to this group, but were not examined in this study. Additionally, using adverse childhood socioeconomic risk markers [53] to identify potential intervention points is warranted and the combination of these and ACEs may be useful in identifying how these experiences contribute to adult situations. In addition, future research could usefully explore whether ACEs experienced earlier in childhood have a differential effect compared to those experienced in the teenage years.

## 6. Conclusions

The current study contributes to the literature highlighting the importance of the relationship between ACEs and self-harm which is not accounted for by the occurrence of depression and anxiety. It is unique because of its focus on ACEs in adults in Scotland who have self-harmed. We found that ACEs are common in a sample of adults presenting to hospital following self-harm. There was clear evidence that ACEs are associated with repeat self-harm, with exposure to multiple ACEs being reported by more than 79.1% of those in the repeat self-harm group. As yet we do not fully understand the exact mechanism through which exposure to ACEs influences repeat self-harm in adulthood, however, identifying and targeting ACEs may provide opportunities to intervene and reduce self-harm and suicide risk.

## Figures and Tables

**Table 1:** Descriptive statistics and univariate binary logistic regression analyses showing differences between first-time versus repeat self-harm groups.

Variable	Total	First	Repeat	Odds Ratio	95% Confidence Intervals	*p*-Value
	N (%)	N (%)	N (%)			
**Demographics**						
**Age Mean (M) Standard Deviation (SD)**	35.9 (13.04)	37.8 (10.63)	35.4 (13.6)	0.99	0.96–1.01	0.30
**Gender**						
**Male**	60 (32)	19 (47.5)	41 (27.7)	2.36	1.15–4.84	0.02 *
**Female**	128 (68)	21 (52.5)	107 (72.3)			
**Sexual orientation**						
**Heterosexual**	161 (87)	38 (95)	123 (84.8)	3.40	0.76–15.11	0.11
**Gay/lesbian/bisexual/pansexual**	24 (13)	2 (5)	22 (15.2)			
**Ethnicity**						
**White background**	182 (97.8)	40 (100)	142 (97.3)	X^2^ (1, *n* = 186) = 1.12		0.30
**Other background**	4 (2.2)	0	4 (2.7)			
**Relationship status**						
**Single/not married**	149 (78.8)	34 (82.9)	115 (77.7)	1.39	0.57–3.43	0.47
**Married/civil partnership**	40 (21.2)	7 (17.1)	33 (22.3)			
**Employment status**						
**Employed (vs. inactive)**	68 (36)	15 (36.6)	53 (35.8)	0.57	0.23–1.43	0.24
**Unemployed (vs. employed)**	33 (17.5)	11 (26.8)	22 (14.9)	0.41	0.17–1.02	0.06
**Inactive (vs. unemployed)**	88 (46.5)	15 (36.6)	73 (49.3)	0.73	0.33–1.61	0.43
**Education**						
**No qualifications (vs. further)**	36 (19.2)	9 (22.5)	27 (18.4)	1.15	0.46–2.89	0.77
**High school qualifications (vs. none)**	80 (42.8)	18 (45)	62 (42.2)	0.77	0.35–1.72	0.53
**Further education (vs. none)**	71 (38)	13 (32.5)	58 (39.5)	0.67	0.26–1.77	0.42
**Current living situation**						
**Alone**	74 (39.2)	13 (31.7)	61 (41.2)	1.5	0.72–3.15	0.27
**With someone**	115 (60.8)	28 (68.3)	87 (58.8)			
**Intent to die (indexed self-harm episode)**						
**No/don’t know**	65 (34.4)	21 (51.2)	44 (29.7)	2.5	1.22–5.03	0.01 *
**Yes**	124 (65.6)	20 (48.8)	104 (70.3)			
**No. of previous self-harm episodes ^a^**						
**1–2**	25 (17)	-	25 (17)			
**3–4**	45 (30.6)	-	45 (30.6)			
**5+**	77 (54.4)	-	77 (54.4)			

^a^ Number of previous suicide attempts and non-suicidal self-injury (NSSI) episodes * *p* < 0.05; ** *p* < 0.01. OR: odds ratio; CI: confidence interval; M: mean.

**Table 2:** Univariate binary logistic regression analyses showing differences between first-time versus repeat self-harm groups on psychosocial measures

Variable	Total	First	Repeat	Odds Ratio	95% Confidence Intervals	*p*-Value
	N (%)	N (%)	N (%)			
Adverse childhood experiences (ACEs)						
Verbal/fear of physical abuse	94 (49.7)	13 (33)	81 (55.1)	2.55	1.22–5.33	0.013 *
Physical abuse	88 (46.6)	11 (26.8)	77 (52)	2.95	1.38–6.34	0.005 *
Sexual abuse	57 (30.5)	6 (15)	51 (34.7)	3.01	1.19–7.65	0.020 *
Emotional neglect	102 (54.3)	14 (34.1)	88 (59.9)	2.88	1.39–5.94	0.004 **
Neglect	49 (25.9)	9 (22)	40 (27)	1.32	0.58–3.00	0.51
Parental separation	95 (50.8)	23 (56.1)	72 (49.3)	0.76	0.38–1.53	0.44
Maternal abuse	69 (36.5)	12 (29.3)	57 (38.5)	1.51	0.72–3.20	0.28
Substance abuse in house	93 (49.2)	17 (41.5)	76 (51.4)	1.45	0.74–3.00	0.26
Mental ill health in house	89 (47.3)	13 (31.7)	76 (51.4)	2.3	1.10–4.79	0.03 *
Family member sent to prison	50 (26.7)	7 (17.1)	43 (29.1)	1.99	0.82–4.83	0.13
No of ACEs experienced 0–3	83 (43.9)	27 (65.9)	56 (37.8)	3.17	1.53–6.55	0.002 **
No of ACEs experienced 4+	106 (56.1)	14 (34.1)	92 (62.2)			
ACE Total M (SD)	4.2 (2.8)	3.05 (2.52)	4.47 (2.75)	1.22	1.06–1.40	0.004 **
Depression M (SD)	19.6 (5.77)	16.7 (6.09)	20.4 (5.44)	1.11	1.05–1.17	0.001 **
Anxiety M (SD)	15.4 (4.64)	14 (4.78)	15.8 (4.53)	1.08	1.01–1.16	0.026 *
Attachment Total M (SD)	50.4 (7.55)	53.3 (7.91)	49.5 (7.26)	0.94	0.89–0.98	0.006 **
Close	17.3 (3.22)	18 (2.72)	17.1 (3.33)	0.91	0.82–1.02	0.09
Dependent	15.7 (5.49)	18.1 (5.06)	15.1 (5.44)	0.95	0.85–0.97	0.002 **
Anxiety	17.3 (4.38)	17.2 (5.19)	17.4 (4152)	1.01	0.933–1.09	0.81

* *p* < 0.05; ** *p* < 0.01

**Table 3:** Multivariate regression analysis of factors distinguishing those reporting a first episode of self-harm with those with repeat self-harm.

Model Variable	Odds Ratio	95% Confidence Intervals	*p*-Value
Depression	1.10	1.00–1.21	0.048 *
Anxiety	0.95	0.85–1.07	0.389
Gender	2.2	1.0–4.83	0.05 *
Dependent attachment	0.95	0.88–1.03	0.233
Intention to die	1.99	0.87–4.48	0.095
Binary ACEs (0–3 versus 4+)	2.4	1.05–5.40	0.038 *

* *p* < 0.05.

## References

[1] World Health Organization (2014). Preventing Suicide. CMAJ.

[2] Information Services Division (2016). A Profile of Deaths by Suicide in Scotland 2009–2014: A Report from the Scottish Suicide Information Database.

[3] O’Connor R.C., Nock M.K. (2014). The Psychology of Suicidal Behaviour. Lancet Psychiatry.

[4] O’Connor R.C. (2011). The integrated motivational-volitional model of suicidal behavior. Crisis.

[5] Franklin J.C., Ribeiro J.D., Fox K.R., Bentley K.H., Kleiman E.M., Huang X., Musacchio K.M., Jaroszewski A.C., Chang B.P., Nock M.K. (2017). Risk factors for suicidal thoughts and behaviors: A meta-analysis of 50 years of research. Psychol. Bull..

[6] O’Connor R.C., Kirtley O.J. The integrated motivational-volitional model of suicidal behaviour. Philos. Trans. R. Soc. B..

[7] Arensman E., Griffin E., Corcoran P. (2016). Self-Harm. The International Handbook of Suicide Prevention.

[8] Chan M.K.Y., Bhatti H., Meader N., Stockton S., Evans J., O’Connor R.C., Kapur N., Kendall T. (2016). Predicting suicide following self-harm: Systematic review of risk Factors and risk scales. Br. J. Psychiatry.

[9] Foster T., Gillespie K., McClelland R., Patterson C. (1999). Risk factors for suicide independent of DSM-III-R axis I disorder. Case-control psychological autopsy study in Northern Ireland. Br. J. Psychiatry.

[10] Owens D., Horrocks J., House A. (2002). Fatal and Non-Fatal Repetition of Self-Harm. Systematic Review. Br. J. Psychiatry.

[11] Coope C., Donovan J., Wilson C., Barnes M., Metcalfe C., Hollingworth W., Kapur N., Hawton K., Gunnell D. (2015). Characteristics of People Dying by Suicide after Job Loss, Financial Difficulties and Other Economic Stressors during a Period of Recession (2010–2011): A Review of Coroners’ Records. J. Affect. Disord..

[12] Carroll R., Metcalfe C., Gunnell D. (2014). Hospital Presenting Self-Harm and Risk of Fatal and Non-Fatal Repetition: Systematic Review and Meta-Analysis. PLoS ONE.

[13] National Institute for Health and Care Excellence (NICE) Self-Harm: Longer Term Management. www.nice.org.uk/guidance/cg133.

[14] National Institute for Health and Care Excellence (NICE) (2004). Self-Harm. The Short-Term Physical and Psychological Management and Secondary Prevention of Selfharm in Primary and Secondary Care.

[15] Hawton K., van Heeringen K. (2009). Suicide. Lancet.

[16] Cooper J., Kapur N., Webb R., Lawlor M., Guthrie E., Mackway-Jones K., Appleby L. (2005). Suicide after Deliberate Self-Harm: A 4-Year Cohort Study. Am. J. Psychiatry.

[17] Felitti V.J., Anda R.F., Nordenberg D., Williamson D.F., Spitz A.M., Edwards V., Koss M.P., Marks J.S. (1998). Relationship of Childhood Abuse and Household Dysfunction to Many of the Leading Causes of Death in Adults. The Adverse Childhood Experiences (ACE) Study. Am. J. Prev. Med..

[18] Bellis M.A., Lowey H., Leckenby N., Hughes K., Harrison D. (2014). Adverse Childhood Experiences: Retrospective Study to Determine Their Impact on Adult Health Behaviours and Health Outcomes in a UK Population. J. Public Health.

[19] Kelly-Irving M., Lepage B., Dedieu D., Bartley M., Blane D., Grosclaude P., Lang T., Delpierre C. (2013). Adverse Childhood Experiences and Premature All-Cause Mortality. Eur. J. Epidemiol..

[20] Hughes K., Lowey H., Quigg Z., Bellis M.A. (2016). Relationships between Adverse Childhood Experiences and Adult Mental Well-Being: Results from an English National Household Survey. BMC Public Health.

[21] Corcoran P., Gallagher J., Keeley H.S., Arensman E., Perry I.J. (2006). Adverse Childhood Experiences and Lifetime Suicide Ideation: A cross-sectional study in a non-psychiatric hospital setting. Irish Med. J..

[22] Dong M., Anda R.F., Dube S.R., Giles W.H., Felitti V.J. (2003). The Relationship of Exposure to Childhood Sexual Abuse to Other Forms of Abuse, Neglect, and Household Dysfunction during Childhood. Child Abuse Negl..

[23] Ainsworth M.D.S. (1985). Attachments across the Life Span. Bull. N. Y. Acad. Med..

[24] Hazan C., Shaver P.R. (1994). Attachment as an Organizational Framework for Research on Close Relationships. Psychol. Inq..

[25] Bowlby J. (1969). Attachment and Loss: Attachment.

[26] Raby K.L., Labella M.H., Martin J., Carlson E.A., Roisman G.I. (2017). Childhood Abuse and Neglect and Insecure Attachment States of Mind in Adulthood: Prospective, Longitudinal Evidence from a High-Risk Sample. Dev. Psychopathol..

[27] Baer J., Martinez C.D. (2006). Child Maltreatment and Insecure Attachment: A Meta-Analysis. J. Reprod. Infant Psychol..

[28] Kharsati N., Bhola P. (2016). Self-Injurious Behavior, Emotion Regulation, and Attachment Styles among College Students in India. Ind. Psychiatry J..

[29] Joiner T.E.J., Sachs-Ericsson N.J., Wingate L.R., Brown J.S., Anestis M.D., Selby E.A. (2007). Childhood Physical and Sexual Abuse and Lifetime Number of Suicide Attempts: A Persistent and Theoretically Important Relationship. Behav. Res. Ther..

[30] Kalmakis K.A., Chandler G.E. (2015). Health Consequences of Adverse Childhood Experiences: A Systematic Review. J. Am. Assoc. Nurse Pract..

[31] Corcoran A., Adverse P.I. (2006). Title Adverse Childhood Experiences and Lifetime Suicide Ideation: A Cross-Sectional Study in a Non-Psychiatric Hospital Setting Childhood Experiences and Lifetime Suicide Ideation: A Cross-Sectional Study in a Non-Psychiatric Hospital Setting. Ir. Med. J..

[32] O’Connor D.B., Green J.A., Ferguson E., O’Carroll R.E., O’Connor R.C. (2018). Effects of Childhood Trauma on Cortisol Levels in Suicide Attempters and Ideators. Psychoneuroendocrinology.

[33] Walsh D., McCartney G., Collins C., Taulbut M., Batty G.D. (2017). History, Politics and Vulnerability: Explaining Excess Mortality in Scotland and Glasgow. Public Heatlth.

[34] Office of National Statistics Suicides in the UK: 2016 registrations. https://www.ons.gov.uk/peoplepopulationandcommunity/birthsdeathsandmarriages/deaths/bulletins/suicidesintheunitedkingdom/2016registrations.

[35] O’Connor R.C., Wetherall K., Cleare S., Eschle S., Drummond J., Ferguson E., O’Connor D.B., O’Carroll R.E. (2018). Suicide Attempts and Non-Suicidal Self-Harm: A National Prevalence Study of Young Adults. BJPsych Open.

[36] Armitage C.J., Rahim W.A., Rowe R., O’Connor R.C. (2016). An Exploratory Randomised Trial of a Simple, Brief Psychological Intervention to Reduce Subsequent Suicidal Ideation and Behaviour in Patients Admitted to Hospital for Self-Harm. Br. J. Psychiatry.

[37] Silverman M.M., O’Connor R., Pirkis J. (2016). Challenges to Defining and Classifying Suicide and Suicidal Behaviors. The International Handbook of Suicide Prevention.

[38] National Collaborating Centre for Mental Health (2012). Self-Harm: The Longer-Term Management of Self-Harm.

[39] O’Connor R.C., Smyth R., Williams J.M.G. (2015). Intrapersonal Positive Future Thinking Predicts Repeat Suicide Attempts in Hospital-Treated Suicide Attempters. J. Consult. Clin. Psychol..

[40] Kroenke K., Spitzer R.L., Williams J.B. (2001). The PHQ-9: Validity of a Brief Depression Severity Measure. J. Gen. Intern. Med..

[41] Moriarty A.S., Gilbody S., McMillan D., Manea L. (2015). Screening and Case Finding for Major Depressive Disorder Using the Patient Health Questionnaire (PHQ-9): A Meta-Analysis. Gen. Hosp. Psychiatry.

[42] Spitzer R.L., Kroenke K., Williams J.B.W., Lowe B. (2006). A Brief Measure for Assessing Generalized Anxiety Disorder: The GAD-7. Arch. Intern. Med..

[43] Lowe B., Decker O., Muller S., Brahler E., Schellberg D., Herzog W., Herzberg P.Y. (2008). Validation and Standardization of the Generalized Anxiety Disorder Screener (GAD-7) in the General Population. Med. Care.

[44] Collins N.L. (1996). Working Models of Attachment: Implications for Explanation, Emotion and Behavior. J. Pers. Soc. Psychol..

[45] Goldman G.A., Anderson T. (2007). Quality of Object Relations and Security of Attachment as Predictors of Early Therapeutic Alliance. J. Couns. Psychol..

[46] Bellis M.A., Hardcastle K., Ford K., Hughes K., Ashton K., Quigg Z., Butler N. (2017). Does Continuous Trusted Adult Support in Childhood Impart Life-Course Resilience against Adverse Childhood Experiences—A Retrospective Study on Adult Health-Harming Behaviours and Mental Well-Being. BMC Psychiatry.

[47] Bellis M.A., Hughes K., Leckenby N., Hardcastle K.A., Perkins C., Lowey H. (2015). Measuring Mortality and the Burden of Adult Disease Associated with Adverse Childhood Experiences in England: A National Survey. J. Public Health.

[48] Office for National Statistics, National Records of Scotland, Northern Ireland Statistics and Research Agency Ethnicity, Identity, Language and Religion.

[49] National Health Service (NHS) Information Services (2017). Mental Health, Alcohol & Drug Recovery Services—Quarterly Trend Report Notes.

[50] Finkelhor D., Shattuck A., Turner H., Hamby S. (2013). Improving the Adverse Childhood Experiences Study Scale. JAMA Pediatr..

[51] Wade R., Cronholm P.F., Fein J.A., Forke C.M., Davis M.B., Harkins-Schwarz M., Pachter L.M., Bair-Merritt M.H. (2016). Household and Community-Level Adverse Childhood Experiences and Adult Health Outcomes in a Diverse Urban Population. Child Abuse Negl..

[52] Mersky J.P., Janczewski C.E., Topitzes J. (2017). Rethinking the Measurement of Adversity: Moving Toward Second-Generation Research on Adverse Childhood Experiences. Child Maltreatment.

[53] Taylor-Robinson D.C., Straatmann V.S., Whitehead M. (2018). Comment Adverse Childhood Experiences or Adverse Childhood Socioeconomic Conditions?. Lancet Public Health..

